# Maternal varicella antibodies in children aged less than one year: Assessment of antibody decay

**DOI:** 10.1371/journal.pone.0287765

**Published:** 2023-11-10

**Authors:** Shelly Bolotin, Stephanie L. Hughes, Rachel D. Savage, Elizabeth McLachlan, Alberto Severini, Callum Arnold, Susan Richardson, Natasha S. Crowcroft, Shelley Deek, Scott A. Halperin, Kevin A. Brown, Todd Hatchette, Selma Osman, Jonathan B. Gubbay, Michelle Science

**Affiliations:** 1 Public Health Ontario, Toronto, Ontario, Canada; 2 Dalla Lana School of Public Health, University of Toronto, Toronto, Ontario, Canada; 3 Centre for Vaccine Preventable Diseases, University of Toronto, Toronto, Ontario, Canada; 4 Department of Laboratory Medicine and Pathobiology, University of Toronto, Toronto, Ontario, Canada; 5 National Microbiology Laboratory, Public Health Agency of Canada, Ottawa, Ontario, Canada; 6 Department of Medical Microbiology, University of Manitoba, Manitoba, Canada; 7 Division of Infectious Diseases, The Hospital for Sick Children, Toronto, Ontario, Canada; 8 Division of Microbiology, The Hospital for Sick Children, Toronto, Ontario, Canada; 9 Department of Immunization, Vaccines & Biologicals, World Helath Organization, Geneva, Switzerland; 10 Canadian Center for Vaccinology (CCfV), IWK Health Centre, Nova Scotia Health (NSH), and Dalhousie University, Halifax, Nova Scotia (NS), Canada; 11 Department of Paediatrics, University of Toronto, Toronto, Ontario, Canada; 12 Department of Paediatrics, The Hospital for Sick Children, Toronto, Ontario, Canada; Public Health England, UNITED KINGDOM

## Abstract

**Objectives:**

To investigate maternal antibody levels to varicella in infants <12 months of age in Ontario, Canada.

**Study design:**

In this study, we included specimens from infants <12 months of age, born at ≥37 weeks gestational age, who had sera collected at The Hospital for Sick Children (Toronto, Canada) between 2014–2016. We tested sera using a glycoprotein-based enzyme-linked immunosorbent assay (gpELISA). We measured varicella susceptibility (antibody concentration <150mIU/mL) and mean varicella antibody concentration, and assessed the probability of susceptibility and concentration between one and 11 months of age using multivariable logistic regression and Poisson regression.

**Results:**

We found that 32% of 196 included specimens represented infants susceptible to varicella at one month of age, increasing to nearly 80% at three months of age. At six months of age, all infants were susceptible to varicella and the predicted mean varicella antibody concentration declined to 62 mIU/mL (95% confidence interval 40, 84), well below the threshold of protection.

**Conclusions:**

We found that varicella maternal antibody levels wane rapidly in infants, leaving most infants susceptible by four months of age. Our findings have implications for the timing of first dose of varicella-containing vaccine, infection control measures, and infant post-exposure prophylaxis recommendations.

## Introduction

Varicella-zoster virus (VZV or human herpesvirus 3) causes chickenpox as a primary infection, and can reactivate from the ganglionic neurons after a variable latency period as herpes zoster, commonly known as shingles [1]. In temperate climates and in the absence of vaccination programs, varicella incidence is highest in children under 10 years of age, with over 90% of children infected before adolescence [2]. VZV is highly transmissible, and is most infectious 1–2 days prior to the onset of rash [2]. The household secondary attack rate in susceptible individuals ranges from 61–100% in temperate climates [2]. Infections during early childhood are usually self-limiting and generally lead to life-long immunity [3], but complications including bacterial skin infections, pneumonia, febrile convulsions, and encephalitis can occur, with young children being the most vulnerable [4, 5].

Prior to routine vaccination, varicella incidence in Canada was estimated at 350,000 cases per year (11.7 per 1,000 population)[6]. Although authorized in Canada in 1998 [7], varicella vaccine was only privately available until it was added to Provincial and Territorial vaccination programs from 2000–2007 [6, 8]. In Ontario, one dose of varicella vaccine became publicly available for administration at 15 months of age in 2004, with a second dose added in 2011 for children aged four to six years [7]. Individuals born since 2000 are eligible for vaccination. Implementation of a vaccine program in Ontario dramatically decreased reported disease incidence, medically attended disease, and hospitalization compared to the pre-vaccine era, even in age-groups that were not eligible for vaccination [7, 9].

Infants are especially vulnerable to severe disease and complications, and have the highest rate of hospitalization of all age-groups [9, 10]. They can be exposed to varicella in the home, at daycare or playgroups, and in hospital and outpatient healthcare settings [5, 10–12]. Infants receive maternal antibodies transplacentally and, if born to varicella-immune mothers, are protected at birth [13]. However, maternal antibody antibody levels in infants decay over time, leaving them vulnerable to varicella infection in their first year of life [13–15].

The objective of our study was to assess varicella maternal antibody decay in infants in the first year of life in Ontario, Canada.

## Methods

### Population and study design

We included sera collected from infants <12 months between January 1, 2014 and December 31, 2016 as part of clinical testing of infants who were admitted to hospital or seen in the emergency department or outpatient clinics at The Hospital for Sick Children, a large paediatric tertiary care hospital in Toronto, Ontario, Canada. Lists of previously collected available sera were provided from the laboratory at the Hospital for Sick Children in random order, and up to 25 sera were randomly selected from each of eight age-groups: 0 months (0–30 days (d)), 1 month (31–60 d), 2 months (61–89 d), 3 months (90–119 d), 4 months, 5 months, 6–8 months and 9–11 months for serological testing for VZV antibodies. We excluded sera from infants born at <37 weeks gestational age; those with a suspected or confirmed immune deficiency or an underlying condition associated with reduced antibody counts; those that had received intravenous immune globulin, intramuscular immune globulin or blood transfusions; those previously diagnosed with varicella infection; or who have received varicella immunization. We extracted demographic and clinical data on the infants from the hospital patient information system.

### Laboratory testing

We performed serology testing at the National Microbiology Laboratory in Winnipeg, Manitoba, Canada using the VaccZyme glycoprotein IgG enzyme immunoassay (gpELISA) (The Binding Site Group, Birmingham, United Kingdom), which was previously validated by our group [16]. The assay range is 10–810 mIU/mL, but higher values can be extrapolated from the standards. We defined any samples with antibody results of <150 mIU/mL as susceptible, according to the manufacturer’s instructions, based on agreement with time resolved fluorescence immunoassay (TRFIA) and Merck VZV assays [14, 17, 18].

### Epidemiological analysis

We performed all analyses in Stata v12.1. We summarized continuous data using means and standard deviations, or medians and ranges. We used Chi-squared tests to assess the statistical significance of differences between proportions, and a Cochran-Armitage Chi-squared test to test for statistically significant (linear) trends.

To assess the association between infant age and varicella susceptibility, we used multivariable logistic regression with a forward-fitting approach, including infant sex and maternal age *a priori*. We screened other covariates for inclusion using a Chi-squared or Student’s t test if p<0.20. To predict the probability of varicella susceptibility by infant age, we calculated adjusted marginal predictions by infant month of age (as opposed to by age-group, as for the descriptive analysis) using the “margins” command in Stata. We estimated this using the average maternal age for the study, as well as maternal age of 25, 30, 35 and 40 years. To assess model fit, we used the Hosmer-Lemeshow goodness-of-fit test using 5 groups.

To model mean varicella antibody concentration by infant age, we used multivariable Poisson regression with robust standard errors, using a forward-fitting approach as described previously [19]. To address the non-linear relationship between infant age and antibody concentration, we modeled infant age using restricted cubic spline terms with five knots placed at the 5^th^ (age 0.4 months), 27.5^th^ (2.1 months), 50^th^ (4.0 months), 72.5^th^ (5.7 months), and 95^th^ percentiles (11.0 months). To predict mean antibody concentration by infant age, we calculated the adjusted marginal predictions of infant mean varicella antibody concentrations using the average maternal age for the study, as well as maternal age of 25, 30, 35 and 40 years.

To address missing data that were not available in the hospital’s patient information system, we used single random regression imputation to fill in missing data in the maternal age and breastfeeding status variables under the missing-at-random assumption. We performed a sensitivity analysis restricting observations only to infants who had complete data for these predictors (i.e.–complete case analysis) for both models. We presented all estimates using 95% confidence intervals (95% CI).

### Ethics

The study was approved by The Hospital for Sick Children Research Ethics Board and the Public Health Ontario Ethics Review Board. As part of the Hospital for Sick Children Ethics Board review, we obtained a standard waiver for secondary use of existing biological specimens. This waiver allows for retrospective testing of existing serological samples when the results are unlikely to affect the welfare of persons to whom the information relates, and it is imposible or impracticable to seek content from persons from whom the material was collected. These conditions and all others that satisfy Canada’s Tri-Council Policy Statement on the Ethical Conduct for Research Involving Humans were deemed to have been met.

## Results

We tested 196 sera for our study, representing 187 infants. Nine infants contributed two sera samples collected at two different time points. Of the tested specimens, 56% were from males and 35% were from infants with underlying conditions, which were most commonly either central nervous system conditions/developmental delays, or gastrointestinal/liver conditions (Table 1). Detailed characteristics of the 196 sera can be found in a previous publication [19]. The mean and median maternal age at the time of specimen collection was 32 years (standard deviation 6, range 18–47).

**Table 1:** Characteristics of study population (N = 196).

Variable	Overall (N = 196) n	Susceptible (n = 127) n (%)	Immune (n = 69) n (%)	P-value
Infant age (months)				
0	25	2 (8.0)	23 (92.0)	<0.001
1	25	8 (32.0)	17 (68.0)	
2	24	13 (54.2)	11 (45.8)	
3	24	19 (79.2)	5 (20.8)	
4	24	17 (70.8)	7 (29.2)	
5	25	20 (80.0)	5 (20.0)	
6–8	25	25 (100.0)	0 (0.0)	
9–11	24	23 (95.8)	1 (4.2)	
Mean infant age in months (SD)	4 (3)	6 (3)	2 (2)	<0.0001
Infant sex				
Male	110	70 (63.6)	40 (36.4)	0.70
Female	86	57 (66.3)	29 (33.7)	
Underlying condition				
Yes	69	51 (73.9)	18 (26.1)	0.05
No	127	76 (59.8)	51 (40.2)	
Admission setting				
Inpatient	92	61 (66.3)	31 (33.7)	0.03
Outpatient	93	55 (59.1)	38 (40.9)	
Emergency department	11	11 (100.0)	0 (0.0)	
Gestational age (weeks)^a^				
37	21	14 (66.7)	7 (33.3)	0.22
38	28	21 (75.0)	7 (25.0)	
39	35	19 (54.3)	16 (45.7)	
40	21	10 (47.6)	11 (52.4)	
≥41	24	17 (70.8)	7 (29.2)	
Any breast feeding^b^				
Yes	79	44 (55.7)	35 (44.3)	0.98
No	25	14 (56.0)	11 (44.0)	
Mean maternal age^c^ in years (SD, range)	32 (6, 18–47)	32 (6, 18–45)	33 (6, 22–47)	0.48
GMC (mIU/mL)	95	39	488	

SD, standard deviation. GMC, geometric mean concentration.

A—excludes 67 missing; b—excludes 92 missing; c—excludes 96 missing.

### Infant varicella susceptibility

The proportion of samples from infants susceptible to varicella increased with each increasing infant month of age (p<0.001, Cochran-Armitage test for trend p<0.0001) (Table 1). Before one month of age, 8% (2/25) were susceptible to varicella, increasing to 54.2% (13/24) by two months of age, and to 100% by six months of age. Infant susceptibility was not found to differ significantly by sex (p = 0.70), gestational age (p = 0.22), breastfeeding status (p = 0.98), or by mean maternal age (p = 0.48). A higher proportion of infants with an underlying condition were susceptible to varicella compared to those without such conditions (73.9% or 51/69 vs. 59.8% or 76/127, p = 0.05). There was a statistically significant difference between the proportion of samples from susceptible infants who were inpatients (66.3% or 61/92), outpatients (59.1% or 55/93) or those seen in the emergency department (100% or 11/11) (p = 0.03).

### Predictors of infant varicella susceptibility

For each month increase in infant age, the odds of varicella susceptibility nearly doubled (unadjusted odds ratio (OR) = 1.98, 95% CI 1.61, 2.43). After forward stepwise logistic regression, no other variables were eligible for inclusion other than those selected *a priori* (Table 2). After adjusting for infant sex and maternal age (with imputation), the association remained unchanged (adjusted OR = 1.99, 95% CI 1.61, 2.45). Results were similar when we adjusted for infant sex and maternal age using complete case analysis (n = 100) (adjusted OR = 1.94, 95% CI 1.46, 2.57).

**Table 2:** Crude and adjusted logistic regression analysis of the association between infant age and susceptibility to varicella.

Variable	Unadjusted		Adjusted (Imputed)		Adjusted (Complete-case)	
	(N = 196)		(N = 196)		(N = 100)	
	OR	95% CI	OR	95% CI	OR	95% CI
Infant age (per 1-month increase)	1.98	1.61, 2.43	1.99	1.61, 2.45	1.94	1.46, 2.57
Maternal age (per 1-year increase)	—	—	0.97	0.92, 1.04	0.96	0.87, 1.05
Infant sex (female)	—	—	0.92	0.43, 1.95	0.92	0.32, 2.59

**Fig 1:**
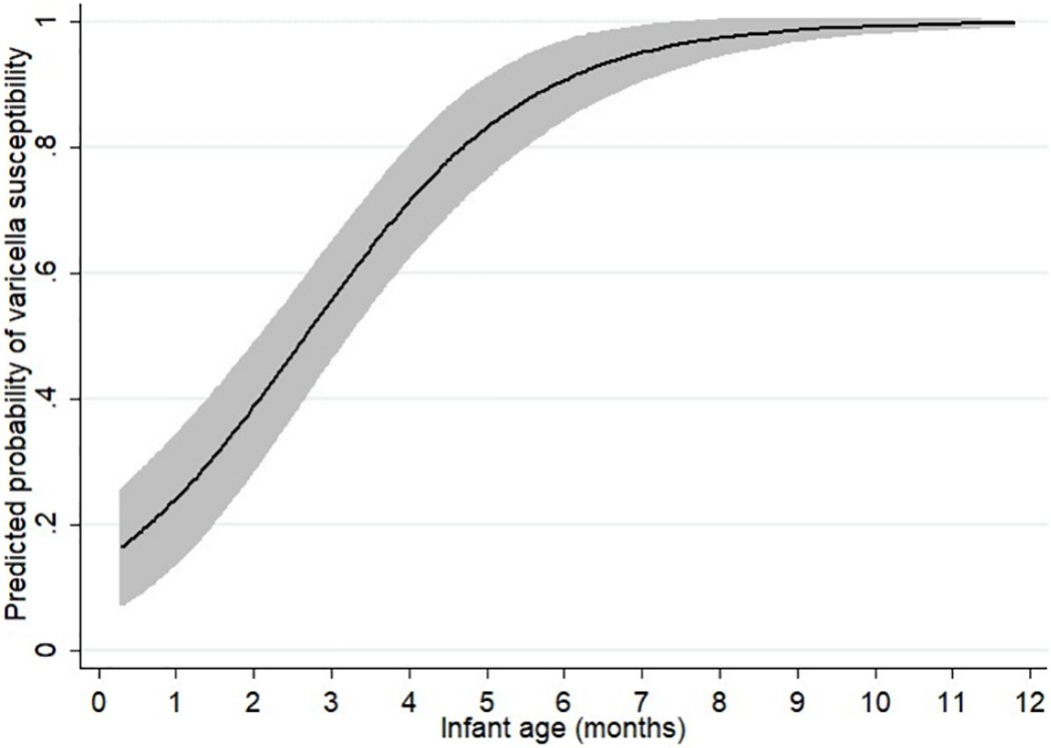
Predicted probability of varicella susceptibility by infant age, for mothers aged 32 years, based on a logistic model controlling for infant sex and maternal age. The shaded area represents 95% CIs.

We predicted the probability of infant varicella susceptibility by age, using the mean maternal age in the study (32 years) and controlling for infant sex. We found that the probability of susceptibility was 24% (95% CI 14%, 34%) at one month of age, increasing to 71% (95% CI 63%, 80%) by four months and to 97% (95% CI 95%, 100%) at eight months (Fig 1, S1 Table).

We observed a modest inverse relationship between maternal age and the probability of infant susceptibility (S1 Fig). For example, the probability of varicella susceptibility in a one month old infant was approximately 20% if their mother was 40 years of age, but almost 30% if their mother was 25 years of age. However, maternal age did not significantly contribute to the odds of susceptibility in our logistic regression model (Table 2).

### Infant varicella antibody levels

**Fig 2:**
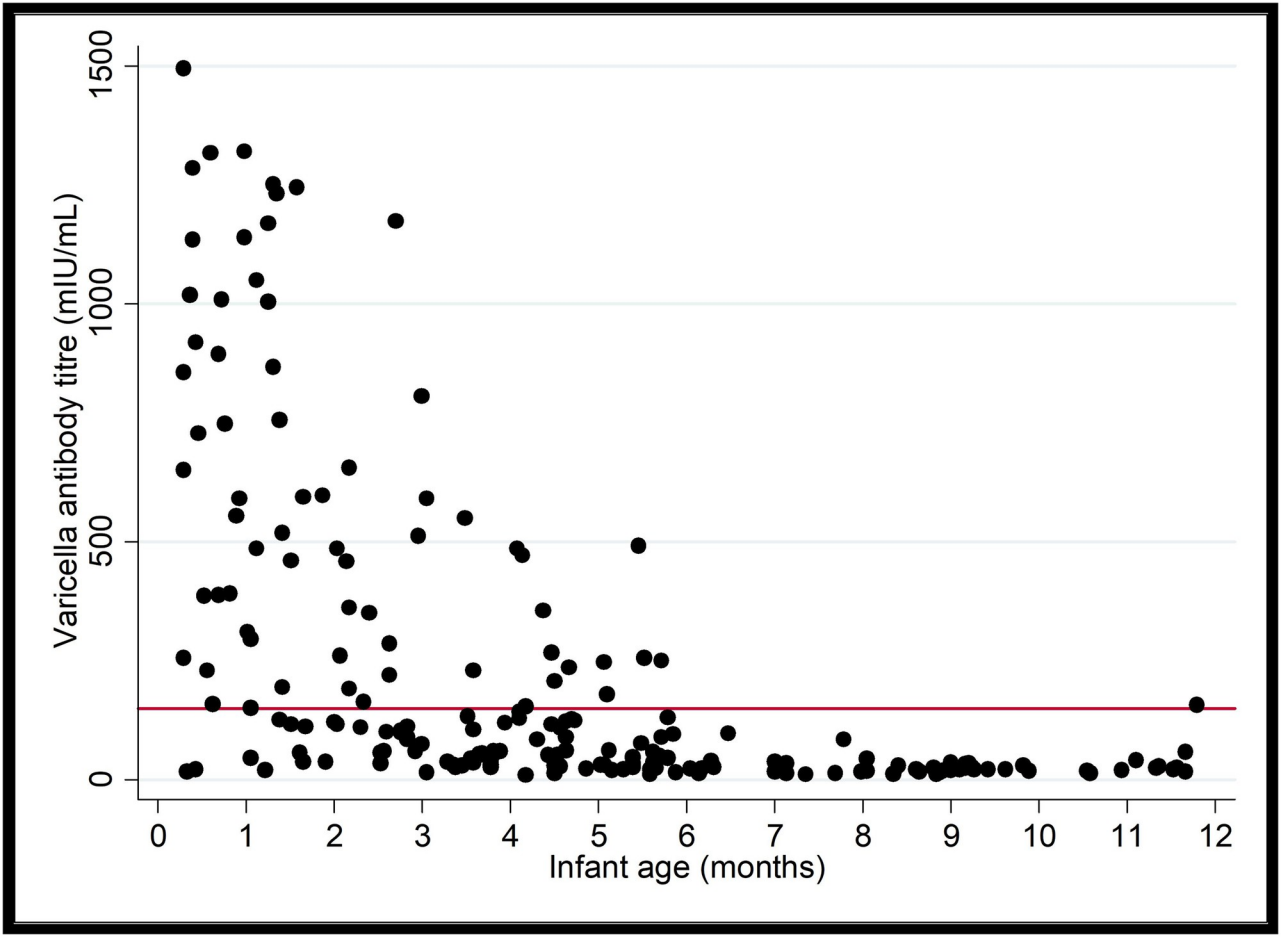
Scatterplot of the relationship between infant age in months and their varicella antibody concentration in mIU/mL (N = 196). We considered infants above the threshold (red line, 150mIU/mL) to be immune to varicella infection, and those below the threshold to be susceptible to varicella infection.

Varicella antibody concentrations at birth varied greatly, ranging from concentrations under the threshold of protection to very high concentrations >1000 mIU/mL (Fig 2). Controlling for infant sex and maternal age and using the average maternal age in the study (32 years), the predicted mean varicella antibody concentration in infants one month and four months of age was 584 mIU/mL (95% CI 471, 698) and 135 mIU/mL (95% CI 88, 182), respectively (S2 Fig, S1 Table). By six months of age, the predicted mean varicella antibody concentration declined to 62 mIU/mL (95% CI 40, 84), well below the threshold of 150 mIU/mL.

We found that infant antibody concentrations increased slightly with maternal age (S3 Fig), although overall predicted concentrations were similar across the range of maternal ages.

## Discussion

In this study of 196 infant sera, we found that maternally acquired antibodies to varicella waned rapidly in the first 12 months of life. More than half of the specimens from infants two months of age and all specimens from infants six months of age were found to be susceptible to varicella. Our findings highlight a large gap in protection between the time when infants lose maternal antibody-mediated protection and eligibility for a first varicella vaccine dose, which occurs at either 12 or 15 months of age (depending on the province/territory) in Canada, at 12–15 months in the United States, and at 18 months in Australia [7, 20–22]. This susceptibility gap is similar albeit slightly wider than described in studies performed in varicella-endemic settings, in which the majority of infants were susceptible to varicella by 3–6 months of age [23], and all were susceptible by nine months of age [24]. This wide susceptibility gap is concerning because while most varicella infections are self-limiting, case fatality rates in infants are four times higher than in older children [10], and clinical complications upon infection are inversely correlated to varicella antibody levels [24]. As a result, our findings have important implications for post-exposure management of exposed infants. Varicella vaccine is not recommended for administration in infants <12 months in all jurisdictions, and therefore cannot be used for post-exposure prophylaxis [25], and immune globulin is not generally recommended unless the infant has a mother that is infected with varicella within a certain time period [26], the infant is premature and in intensive care, or is immunocompromised [25, 27, 28]. Since post-exposure prophylaxis options are limited, exclusion and isolation protocols are of greater importance to prevent transmission. However, infants less than six months are often assumed to be immune to varicella based on maternal immunity. That assumption could lead to exposures of vulnerable infants, since our findings suggest most are susceptible by four months of age.

Our findings are also important with regards to the timing of the first dose of varicella vaccine, which balances the risk of varicella infection with the presence of maternal antibodies, since the latter can interfere with the infant immune response to vaccine. The duration of infant immunity correlates to maternal antibody concentration [14, 15], but several factors can affect varicella antibody levels in mothers. Decreased circulation of varicella in some jurisdictions [9, 29] may lead to decreased immunological boosting and antibody waning in mothers [30], leading to earlier waning of maternal protection in their infants. Studies have shown mixed results regarding the waning of infection-induced vs. vaccine-induced antibodies against varicella [31] [32–37], and while none of the mothers in this study were vaccinated, the consequence of maternal varicella vaccination on infant immunity requires further study.

### Limitations

Our analysis was limited by inclusion of infants from a single tertiary care pediatric hospital, who represent an urban population. There were missing data on maternal age and breastfeeding status. For the latter, the lack of more detailed data on extent of and duration of breastfeeding is a limination. Our retrospective study design did not allow collection of other maternal data variables, like varicella immunity status or place of birth, or the collection of maternal sera to assess the correlation between infant and maternal antibody levels. Furthermore, the specimens were collected in 2014–2016, at a time when individuals of childbearing age were mostly immune through previous infection, and not vaccination. In future studies, sampling both the mother and infant would capture a more complete picture of maternal risk factors for infant susceptibility. Lastly, although antibody levels generally correlate to immunity [38], this correlation is usually observed in indiviauls that also have cellular immunity to varicella [39]. It is therefore not clear whether all seropositive infants in this study, who have no varicella cellular immunity, are truly protected from infection.

## Conclusions

In summary, our results suggest that parents, clinicians and policy makers should be aware of potential infant susceptibility to varicella. Our findings can inform hospital isolation protocols to limit transmission to vulnerable infants, by ensuring that immunity testing and not age-based considerations determine whether infants are considered protected from varicella. Further research may be required to optimize vaccination schedules, specifically to investigate the effect of age on varicella vaccine effectiveness in order to understand whether earlier administration of the first varicella vaccine dose would affect protection later in life. In the meantime, infant exposure would best be minimized by increasing population immunity to varicella through high vaccine coverage in eligible groups, family members in close contact with infants, and vaccination of susceptible women of childbearing age to reduce the burden of disease in infants as well as congenital varicella syndrome [40].

## Supporting information

S1 Fig Predicted logistic regression probability of varicella susceptibility by infant age, controlling for infant sex and maternal age, for mothers aged 25, 30, 35 and 40 years. (TIF)

S2 Fig Predicted mean varicella antibody concentrations by infant age, controlling for sex, for mothers aged 32 years, based on a Poisson regression model. The shaded area represents 95% CIs.
(TIF)

S3 Fig Predicted poisson regression mean varicella antibody concentrations by infant age, controlling for infant sex and maternal age, for mothers aged 25, 30, 35 and 40 years. (TIF)

S1 Table Predicted probability of infant varicella susceptibility and predicted mean varicella antibody concentration, adjusted for infant sex and maternal age, and assuming a maternal age of 32 years. (DOCX)
