# Supplementary figures and images for: Maternal varicella antibodies in children aged less than one year: Assessment of antibody decay
Source: PLoS One. 2023 Nov 10;18(11):e0287765. doi: 10.1371/journal.pone.0287765 (PMC10637651; doi:10.1371/journal.pone.0287765)

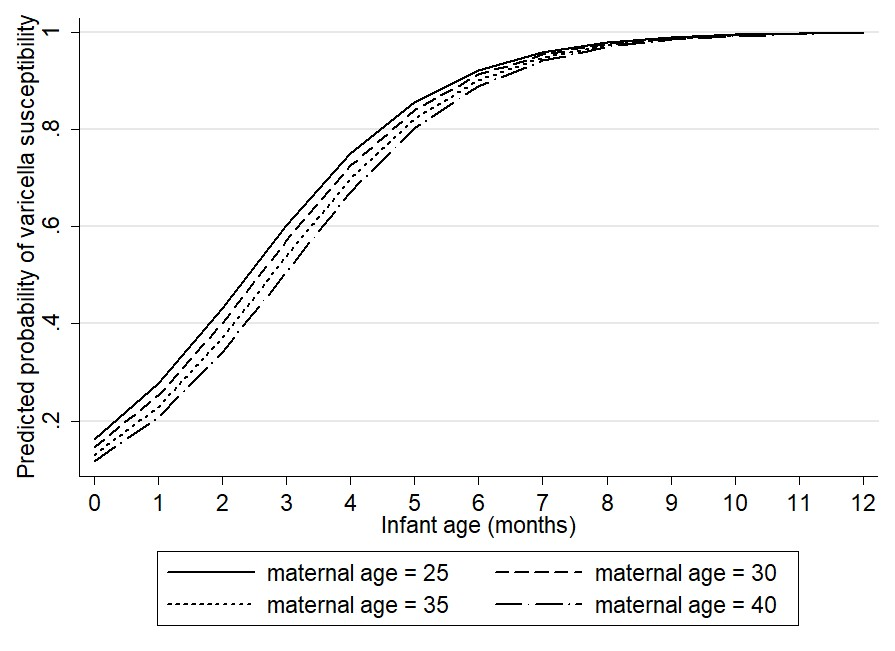

Supplement: S1 Fig — (TIF) [file pone.0287765.s001.tif]

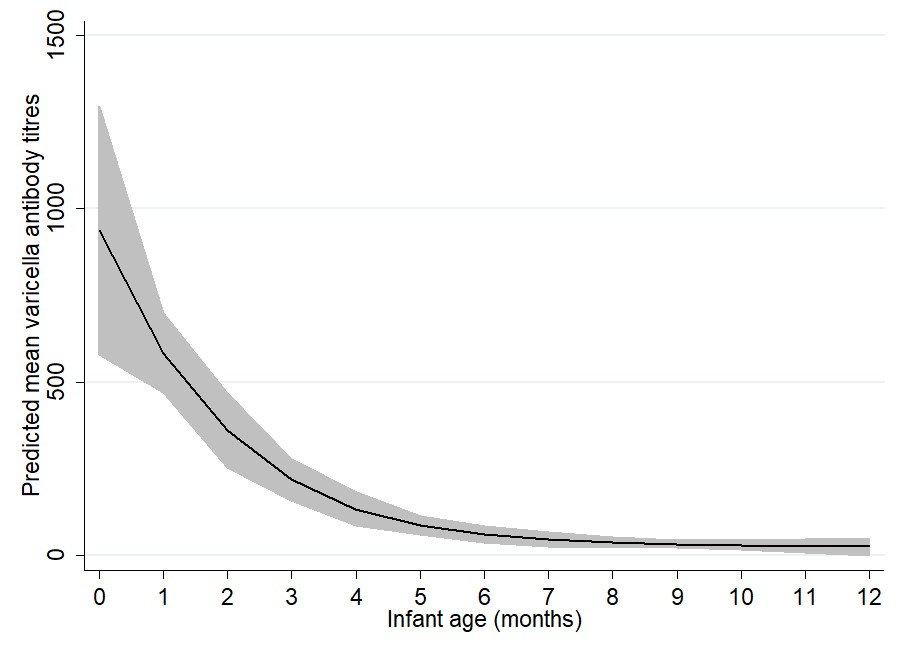

Supplement: S2 Fig — The shaded area represents 95% CIs. (TIF) [file pone.0287765.s002.tif]

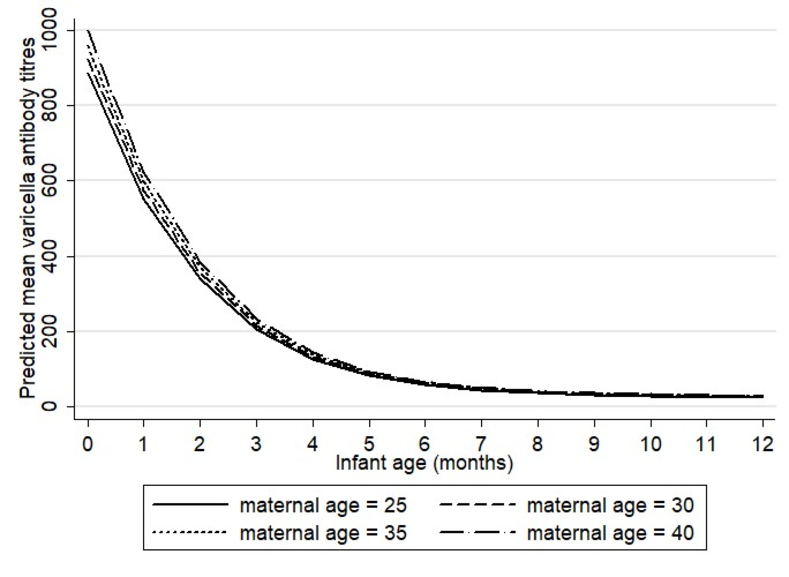

Supplement: S3 Fig — (TIF) [file pone.0287765.s003.tif]
