# Supplementary material for: Maternal varicella antibodies in children aged less than one year: Assessment of antibody decay
Source: PLoS One. 2023 Nov 10;18(11):e0287765. doi: 10.1371/journal.pone.0287765 (PMC10637651; doi:10.1371/journal.pone.0287765)
Supplement: S1 Table — (DOCX) [file pone.0287765.s004.docx]

**S1 Table.**

Predicted probability of infant varicella susceptibility and predicted mean varicella antibody concentration, adjusted for infant sex and maternal age, and assuming a maternal age of 32 years

| Infant age (months) | Predicted probability | 95% confidence interval | Mean concentration (mIU/mL) | 95% confidence interval |
| --- | --- | --- | --- | --- |
| 0 | 14% | 5%, 23% | 936 | 579, 1294 |
| 1 | 24% | 14%, 34% | 584 | 471, 698 |
| 2 | 39% | 29%, 49% | 361 | 255, 467 |
| 3 | 56% | 47%, 65% | 219 | 161, 278 |
| 4 | 71% | 63%, 80% | 135 | 88, 182 |
| 5 | 83% | 75%, 91% | 88 | 62, 113 |
| 6 | 91% | 85%, 97% | 62 | 40, 84 |
| 7 | 95% | 91%, 99% | 48 | 28, 68 |
| 8 | 97% | 95%, 100% | 39 | 25, 54 |
| 9 | 99% | 97%, 100% | 34 | 24, 45 |
| 10 | 99% | 98%, 100% | 31 | 19, 43 |
| 11 | 100% | 99%, 100% | 29 | 10, 47 |
